# Assessment of esophagogastric junction morphology by dynamic real-time MRI: comparison of imaging features to high-resolution manometry

**DOI:** 10.1007/s11604-021-01210-9

**Published:** 2021-12-07

**Authors:** Lorenz Biggemann, Johannes Uhlig, Nina Gliem, Omar Al-Bourini, Edris Wedi, Volker Ellenrieder, Michael Ghadimi, Martin Uecker, Jens Frahm, Joachim Lotz, Ali Seif Amir Hosseini, Ulrike Streit

**Affiliations:** 1 Department of Diagnostic and Interventional Radiology, University Medical Center Göttingen, Göttingen, Germany; 2 Department of Gastroenterology and Gastrointestinal Oncology, University Medical Center Göttingen, Göttingen, Germany; 3 Department of General, Visceral, and Paediatric Surgery, University Medical Center, Göttingen, Germany; 4 Biomedical NMR, Max-Planck-Institute for Biophysical Chemistry, Göttingen, Germany

**Keywords:** Real-time MRI, Esophagogastric junction, EGJ, High-resolution manometry, HRM

## Abstract

**Purpose:**

To assess the esophagogastric junction (EGJ) on real-time MRI and compare imaging parameters to EGJ morphology on high-resolution manometry (HRM).

**Methods:**

A total of 105 of 117 eligible patients who underwent real-time MRI and high-resolution manometry for GERD-like symptoms between 2015 and 2018 at a single center were retrospectively evaluated (male *n* = 57; female *n* = 48; mean age 52.5 ± 15.4 years). Real-time MRI was performed at a median investigation time of 15 min (1 frame/40 ms). On HRM, EGJ morphology was assessed according to the Chicago classification of esophageal motility disorders. Real-time MRI was performed at 3 T using highly undersampled radial fast low-angle shot acquisitions with NLINV image reconstruction. A 10 mL pineapple juice bolus served as oral contrast agent at supine position. Real-time MRI films of the EGJ were acquired during swallowing events and during Valsalva maneuver. Anatomic and functional MRI parameters were compared to EGJ morphology on HRM.

**Results:**

On HRM, *n* = 42 patients presented with EGJ type I (40.0%), *n* = 33 with EGJ type II (31.4%), and *n* = 30 with EGJ type III (28.6%). On real-time MRI, hiatal hernia was more common in patients with EGJ type III (66.7%) than in patients with EGJ type I (26.2%) and EGJ type II (30.3%; *p* < 0.001). Sliding hiatal hernia was more frequent in patients with EGJ type II (33.3%) than in patients with EGJ type III (16.7%) and EGJ type I (7.1%; *p* = 0.017). The mean esophagus–fundus angle of patients was 85 ± 31° at rest and increased to 101 ± 36° during Valsalva maneuver.

**Conclusion:**

Real-time MRI is a non-invasive imaging method for assessment of the esophagogastric junction. Real-time MRI can visualize dynamic changes of the EGJ during swallowing events.

**Supplementary Information:**

The online version contains supplementary material available at 10.1007/s11604-021-01210-9.

## Introduction

Gastroesophageal reflux disease (GERD) is defined as reflux of gastric contents into the distal esophagus, which may yield relevant clinical symptoms or complications [1]. The esophagogastric junction (EGJ) is a pivotal defense mechanism against gastric reflux [2]. Three structures have been identified as integral components of the EGJ: the intrinsic lower esophageal sphincter (LES), the sling fibers of the gastric cardia and the extrinsic crural diaphragm, which together form an intricate sphincter complex [2, 3]. The anatomic conformation of the sling fibers constitutes a flap valve mechanism that improves the barrier function [4, 5]. Transient relaxations of the lower esophageal sphincter complex (TLESRs) have been identified as the main cause of EGJ dysfunction for gastroesophageal reflux [6, 7]. Moreover, hiatal hernias may impair the sphincter function of the crural diaphragm and sling fibers [8, 9]. Currently, EGJ morphology is assessed using high-resolution manometry (HRM). HRM distinguishes between three EGJ types by measuring the extent of separation of pressure peaks between the LES and crural diaphragm [10, 11]. However, HRM does not provide direct anatomic delineation of the esophagogastric sphincter complex.

Real-time MRI enables dynamic visualization of the gastroesophageal junction and the adjacent diaphragm [12, 13]. Pineapple juice serves as an oral contrast agent for real-time MRI to visualize the bolus transit through the EGJ [14]. In previous studies, real-time MRI showed diagnostic potential for detection of fundoplication failure [15]. Therefore, the presence or recurrence of hiatal hernia is one of the most common clinical questions for real-time MRI studies. In previous studies, we have experienced changes of the distance between the LES and diaphragm between resting position and Valsalva maneuver [13]. Still, the clinical relevance of these changes remains unclear, and correlations with HRM as an invasive reference standard are missing.

Our study, therefore, aims to compare real-time MRI findings to EGJ morphology on HRM.

## Methods

### Study population

This retrospective cohort study was conducted in accordance with the Helsinki Declaration in its most recent version und received approval by the local ethics board (NR 14/5/18). Patients were recruited from the Department of General, Visceral, and Paediatric Surgery and the Department of Gastroenterology and Gastrointestinal Oncology of the University Medical Center Göttingen. To be considered for inclusion, patients must have exhibited GERD-like symptoms for at least 6 months and undergone real-time MRI and HRM between 2015 and 2018. Patients were excluded if high-resolution manometry was aborted or could not be evaluated. All participants gave written informed consent before each examination.

### High-resolution manometry

EGJ morphology was assessed by high-resolution manometry. For measurements of pressure plots, a nasogastric catheter was placed into the stomach (Unisensor AG, Attikon, Switzerland). All patients were asked to perform 10 separate swallows of 5 mL of water in an upright position. After correct placement of the catheter, the position of the LES was identified on pressure plots using ViMeDat™ Version 5.0.0.3117 (Standard Instruments GmbH, Karlsruhe, Germany). EGJ morphology was analyzed on pressure tomography plots according to the Chicago classification of esophageal motility disorder, v3.0 [11]: complete overlap of the crural diaphragm and the lower esophageal sphincter on the spatial pressure variation plots was considered EGJ Type I. Double peaked pressure zones with an identifiable separation of the crural diaphragm and the LES > 1 cm and < 2 cm were graded EGJ Type II. A separation of the crural diaphragm and the LES > 2 cm was categorized as EGJ Type III. If possible, EGJ type III was further stratified into two subgroups: EGJ Type IIIa or EGJ Type IIIb, depending on the position of the respiratory inversion point, located either proximal to the crural diaphragm and or proximal to the LES. If no further subclassification was possible, the EGJ Type III was defined as “not further classified”.

### Real-time MRI acquisition

All MRI studies were performed at using a commercial 3 T scanner (Siemens Skyra, Siemens Healthineers, Erlangen, Germany) with an 18-element thorax coil and suitable elements of the spine coil array. Real-time MRI was based on highly undersampled radial fast low-angle shot acquisitions with NLINV image reconstruction [16]. In summary, the real-time MRI used in this study was optimized to visualize pineapple juice passage over the esophagus with temporal resolution of 40 ms. Further technical details have been described previously [15, 17].

Real-time MRI films of the EGJ were obtained with the following parameters: TR = 2.12 ms, TE = 1.31 ms, and flip angle 8°. The use of 19 spokes per frame resulted in a measurement time of 40 ms per frame, the overall resolution, therefore, was 25 frames per second (fps). A field of view of 256 × 256 mm^2^ in conjunction with a data matrix of 170 × 170 yielded an in-plane resolution of 1.5 × 1.5 mm^2^. Slice thickness was 8 mm. Online reconstruction of real-time images was achieved by a highly parallelized version of the NLINV algorithm and its implementation on a computer (sysGen/TYAN Octuple-GPU, 2 × 123 Intel Westmere E5620 processor, 48 GB RAM, Sysgen, Bremen, Germany) with 8 graphical processing units (GPUs, GeForce GTX TITAN, Nvidia, Santa Clara, CA, USA).

Commercially available pineapple juice was employed as an oral contrast agent based on its T1 shortening effect due to high manganese ion concentration. Every swallow event used a 10 mL bolus of pineapple juice that was injected into the patient’s mouth through an infusion tube. All patients underwent real-time MRI were performed in supine position. Patients were given the order to perform a self-controlled swallow by the operator at the beginning of the MRI recording. A single pineapple juice bolus of 10 mL was given for each real-time MRI sequence. Real-time MRI films lasted at least for 25 s. After visualization of the bolus transit through the LES in sagittal and coronal oblique planes, patients were asked to perform Valsalva maneuver during real-time MRI acquisitions by simultaneously tensioning the abdominal muscles and exhaling against a closed mouth. All real-time MRI parameters were assessed as previously described [13, 18]. All MRI films were visually assessed for the presence of hiatal hernia at rest. Furthermore, hiatal hernias that occurred only during Valsalva maneuver but not in a resting position were defined as sliding hernia. The sphincter–diaphragm distance was measured at rest and during Valsalva maneuver as the distance of the LES to the crural diaphragm as the imaging equivalent of the separation of the LES and crural diaphragm on HRM. In case of migration of the LES above the diaphragm, the sphincter–diaphragm distance was given a negative value.

### MR image evaluation

Real-time MRI films of each patient were evaluated by two experienced abdominal radiologists by consensus reading. Both readers each had an overall experience of 3 years in real-time MRI of the EGJ and more than 5 years in abdominal radiology. In case of reader disagreement, all MRI films of the study were re-read in a separate session by both readers for a final verdict. If both readers considered a parameter to be not readable the parameter was marked as “non-evaluable” (NE). For image evaluation, the manufacturer’s software was used (Syngo B17, Siemens Healthineers, Erlangen, Germany).

### Statistical analysis

Continuous variables are given as mean ± standard deviation (SD), and categorical variables as absolute values and percentage. Continuous variables were compared using the non-parametric Wilcoxon rank-sum test, and categorical variables using the Chi-square test. All statistical analyses were performed using R version 3.4.3 and RStudio Version 1.1.414. An alpha level of 0.05 was chosen to indicate statistical significance. All provided *p* values are two-sided.

## Results

### Study population

**Fig. 1:**
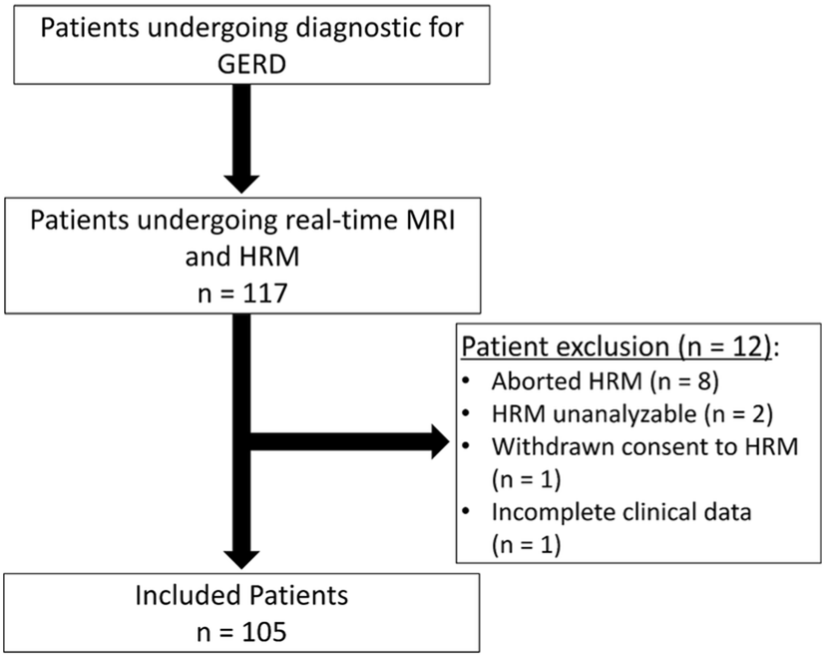
Patient flowchart

Out of 117 eligible patients, a total of 105 met the inclusion criteria. Twelve patients were excluded: *n* = 8 patients aborted HRM, in *n* = 2 cases EGJ type could not be precisely assessed, *n* = 1 patient withdrew consent to HRM, and *n* = 1 case showed insufficient clinical data. Inclusion criteria are depicted in a flowchart in Fig. 1. The mean age of the patients included was 52.5 years (SD: 15.4 years; range 21–87 years). The gender distribution was balanced with 48 females (45.7%) and 57 males (54.3%). There were no differences regarding age (*p* = 0.55) or gender (*p* = 0.77) between different EGJ types. The patient characteristics are summarized in Table 1. All patients underwent real-time MRI without adverse events at a median investigation time of 15 min. No aspiration or allergic reactions to pineapple juice occurred.

**Table 1:** Patient characteristics

	Total *n* = 105	EGJ type 1 *n* = 42	EGJ type 2 *n* = 33	EGJ type 3 *n* = 30	*p* value
Age	52.5 (± 15.4)	48.0 (± 16.3)	53.6 (± 14.7)	57.7 (± 13.3)	0.55
Gender					0.77
Female	48 (45.7%)	21 (50.0%)	14 (42.4%)	13 (43.3%)	
Male	57 (54.3%)	21 (50.0%)	19 (57.6%)	17 (56.7%)	
EGJ type					< 0.001
I	42 (40.0%)	42 (100.0%)	–	–	
II	33 (31.4%)	–	33 (100.0%)	–	
III (not further classified)	7 (6.7%)	–	–	7 (23.3%)	
IIIa	15 (14.3%)	–	–	15 (50.0%)	
IIIb	8 (7.6%)	–	–	8 (26.7%)	

### EGJ assessment by high-resolution manometry

**Fig. 2:**
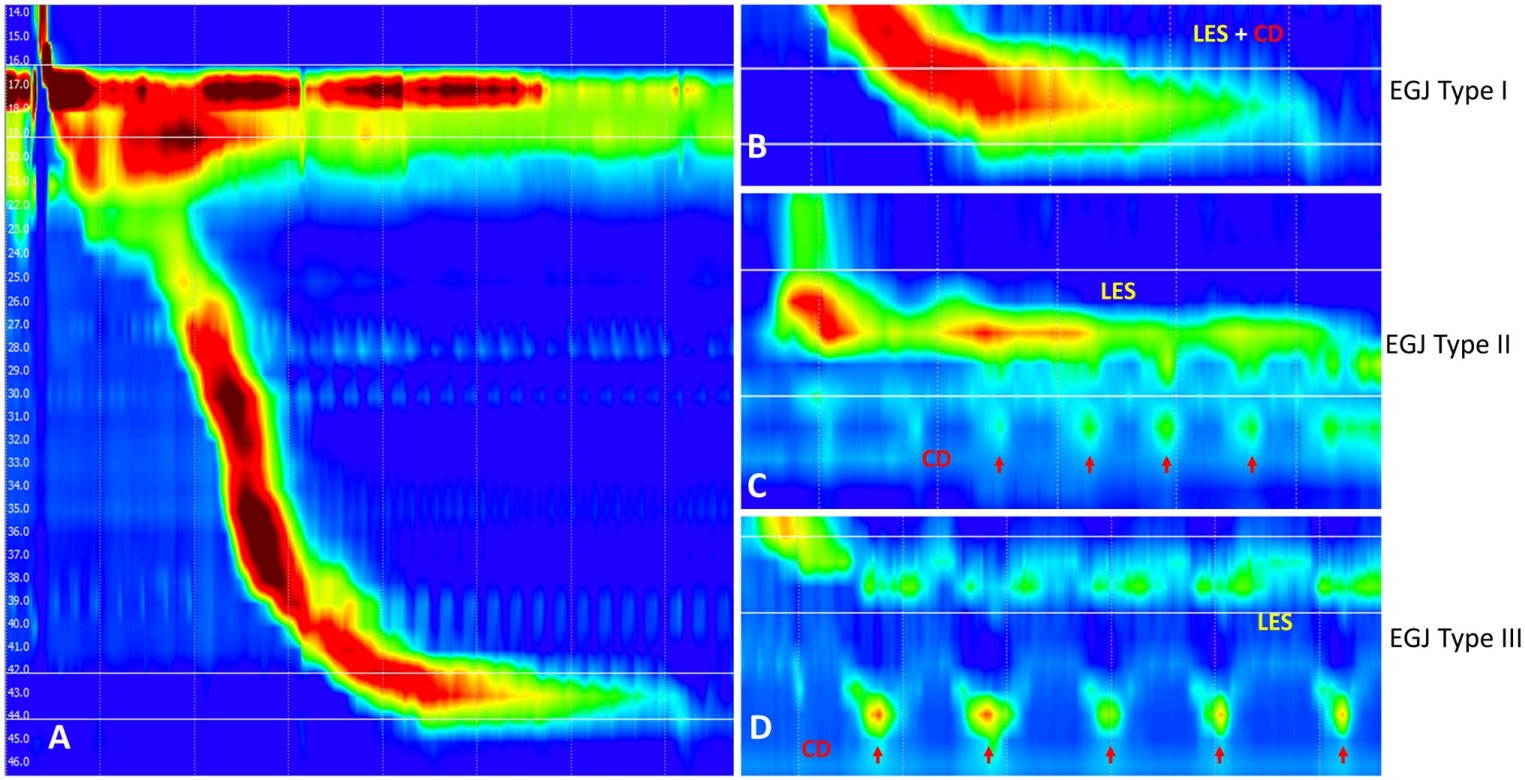
High-resolution manometry visualizes the pressure plots of the swallowing events of a 5 mL water bolus from the upper esophageal sphincter through the esophageal body to the lower esophageal sphincter (LES) (**A**). EGJ type I shows a complete overlap of the pressure plots of the LES and crural diaphragm (CD) (**B**). A separation of the LES and CD > 1 cm and < 2 cm is defined as EGJ type II (**C**) and a separation of > 2 cm as EGJ type III (**D**). All measurements are performed on the scale provided by the software (**A**)

The EGJ type was successfully assessed in *n* = 105 patients on HRM according to the Chicago Classification of esophageal motility disorders, v3.0. A total of 42 patients (40.0%) with complete overlap of the crural diaphragm and the LES were graded as EGJ type I. Furthermore, 33 patients (31.4%) were graded EGJ type II with double peaked pressure zones with a separation of 1–2 cm. Thirty patients (*n* = 30, 28.6%) were assessed with EGJ type III with a separation of the crural diaphragm and LES > 2 cm: *n* = 15 patients were graded as EGJ type IIIa (50.0%), *n* = 8 patients were graded as EGJ type IIIb (26.8%), and *n* = 7 patients were not further classified (23.3%). HRM assessment is visualized in Table 1. Image examples of the 3 different EGJ types are provided in Fig. 2.

### EGJ assessment by real-time MRI

On real-time MRI, hiatal hernias were more common in patients with EGJ type III (*n* = 20; 66.7%) compared to patients with EGJ type I (*n* = 11; 26.2%) and EGJ type II (*n* = 10; 30.3%) (*p* < 0.001). Patients with EGJ type II on HRM had a higher prevalence of sliding hiatal hernia (*n* = 11; 33.3%) compared to patients with EGJ type III (*n* = 5; 16.7%) and EGJ type I (*n* = 3; 7.1%) (*p* = 0.017).

Functional parameters were readable in a total of *n* = 82 (78.1%) patients for sphincter length, *n* = 89 (84.8%) for esophagus–fundus angle at rest, and *n* = 86 (81.9%) for esophagus–fundus angle during Valsalva maneuver. All other functional parameters were readable in > 97.0% of all cases. Real-time MRI parameters are summarized in Table 2.

**Table 2:** MRI parameters

	Total *n* = 105	EGJ type 1 *n* = 42	EGJ type 2 *n* = 33	EGJ type 3 *n* = 30	*p* value
Esophageal diameter					0.66
Mean (SD)	22.0 (± 4.7)	21.2 (± 5.0)	22.8 (± 4.2)	22.2 (± 4.8)	
NE	2 (1.9%)	0 (0%)	0 (0%)	2 (6.7%)	
Sphincter length					0.44
Mean (SD)	17.3 (± 4.1)	17.8 (± 4.6)	18.0 (± 3.9)	15.3 (± 2.6)	
NE	23 (21.9%)	9 (21.4%)	4 (12.1%)	10 (33.3%)	
Sphincter–diaphragm distance (rest)					0.053
Mean (SD)	− 4.7 (± 22.6)	− 1.3 (± 23.3)	0.6 (± 19.1)	− 15.4 (± 22.4)	
NE	3 (2.9%)	2 (4.8%)	0 (0%)	1 (3.3%)	
Sphincter–diaphragm distance (Valsalva)					0.11
Mean (SD)	− 12.4 (± 24.3)	− 6.6 (± 24.6)	− 7.0 (± 21.2)	− 26.2 (± 22.2)	
NE	2 (1.9%)	2 (4.8%)	0 (0%)	0 (0%)	
Change of sphincter–diaphragm distance					0.18
Mean (SD)	− 7.9 (± 12.9)	− 5.3 (± 14.8)	− 7.5 (± 11.2)	− 11.8 (± 11.1)	
NE	3 (2.9%)	2 (4.8%)	0 (0%)	1 (3.3%)	
Sliding hiatal hernia					0.017
Yes	19 (18.1%)	3 (7.1%)	11 (33.3%)	5 (16.7%)	
No	84 (80.0%)	37 (88.1%)	22 (66.7%)	25 (83.3%)	
NE	2 (1.9%)	2 (4.8%)	0 (0.0%)	0 (0.0%)	
Hiatal hernia at rest					< 0.001
Yes	41 (39.0%)	11 (26.2%)	10 (30.3%)	20 (66.7%)	
No	61 (58.1%)	29 (69.0%)	23 (69.7%)	9 (30.0%)	
NE	3 (2.9%)	2 (4.8%)	0 (0.0%)	1 (3.3%)	
Esophagus–fundus angle (rest)					0.47
Mean (SD)	84.9 (± 30.9)	80.1 (± 25.8)	78.3 (± 28.8)	99.4 (± 35.8)	
NE	16 (15.2%)	8 (19.0%)	3 (9.1%)	5 (16.7%)	
Esophagus–fundus angle (Valsalva)					0.41
Mean (SD)	101.1 (± 35.6)	91.3 (± 32.9)	101.7 (± 33.5)	112.8 (± 39.0)	
NE	19 (18.1%)	10 (23.8%)	4 (12.1%)	5 (16.7%)	

The sphincter–diaphragm distance at rest tended to be shorter in patients with EGJ type III (− 15.4 ± 22.4 mm) than in patients with EGJ type I (− 1.3 ± 23.3 mm) and EGJ type II (0.6 ± 19.1 mm), however, differences between groups were not statistically significant (*p* = 0.053). There were no significant differences between different EGJ types regarding the sphincter–diaphragm distance during Valsalva (− 6.6 ± 24.6 vs.

**Fig. 3:**
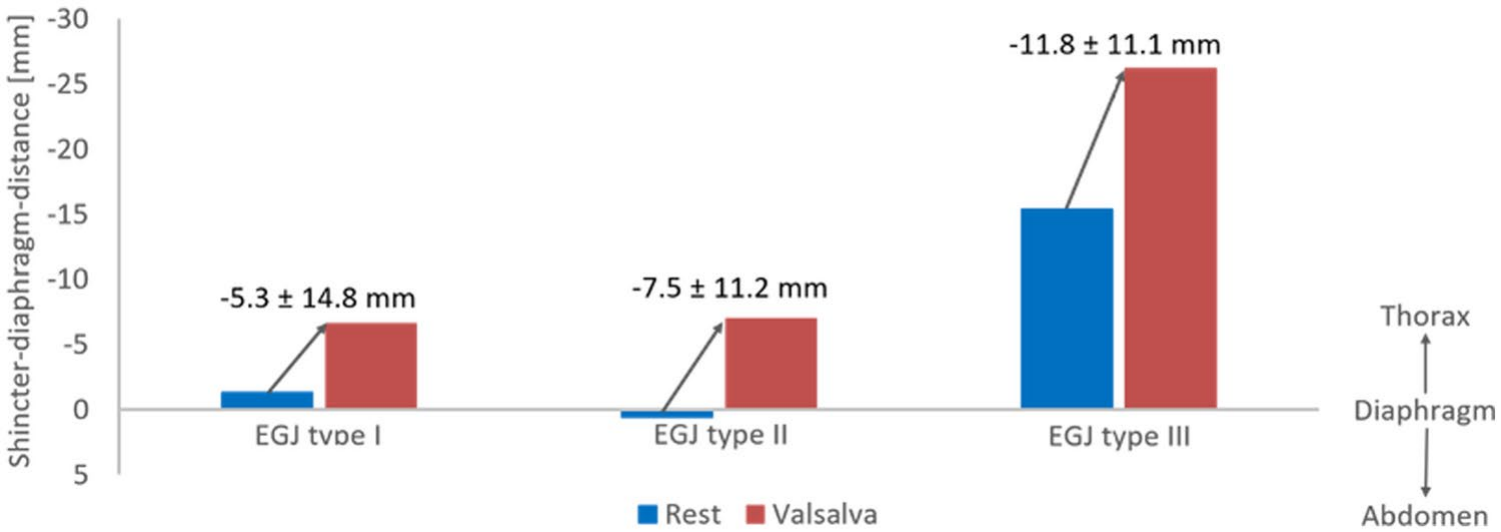
Sphincter–diaphragm distance of different EGJ types on HRM at rest (blue) and during Valsalva (red). The sphincter–diaphragm distance is provided both under resting condition (blue column) and Valsalva maneuver (red column). The change of the sphincter–diaphragm distance is presented as mean ± SD in mm above the columns. Negative values of the sphincter–diaphragm distance indicate position of the LES above the diaphragm

− 7.0 ± 21.2 vs. − 26.2 ± 22.2 mm; *p* = 0.11) or the change of the sphincter–diaphragm distance (− 5.3 ± 14.8 vs. − 7.5 ± 11.2 vs. − 11.8 ± 11.1 mm; *p* = 0.18). The sphincter–diaphragm distances at rest and during Valsalva maneuver are visualized in Fig. 3.

**Fig. 4:**
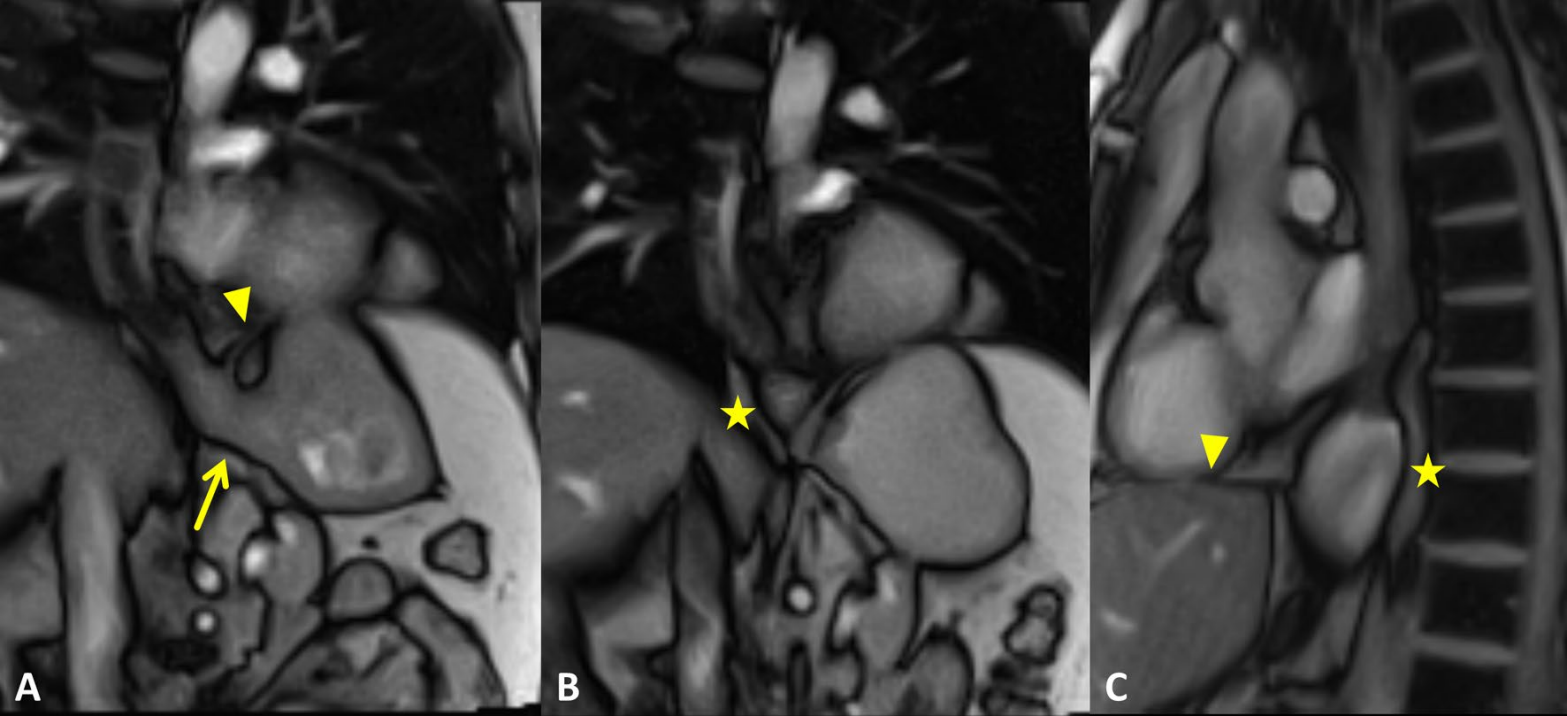
Real-time MRI of the EGJ at rest (**A**) and during Valsalva (**B** and **C**) in a patient with EGJ type I on HRM. MRI performed in coronal oblique planes (**A** and **B**) at sagittal planes (**C**). At rest, the EGJ (arrow) is clearly positioned below the diaphragm (arrowhead) (**A**), confirming the EGJ morphology on HRM. However, the EGJ moved above the diaphragm during Valsalva maneuver (**B**) and formed a small hiatal hernia (large arrow). Repetitive Valsalva maneuver resulted in a relevant size increase of the hiatal hernia (star; **C**). See also supplementary material Video 1

**Fig. 5:**
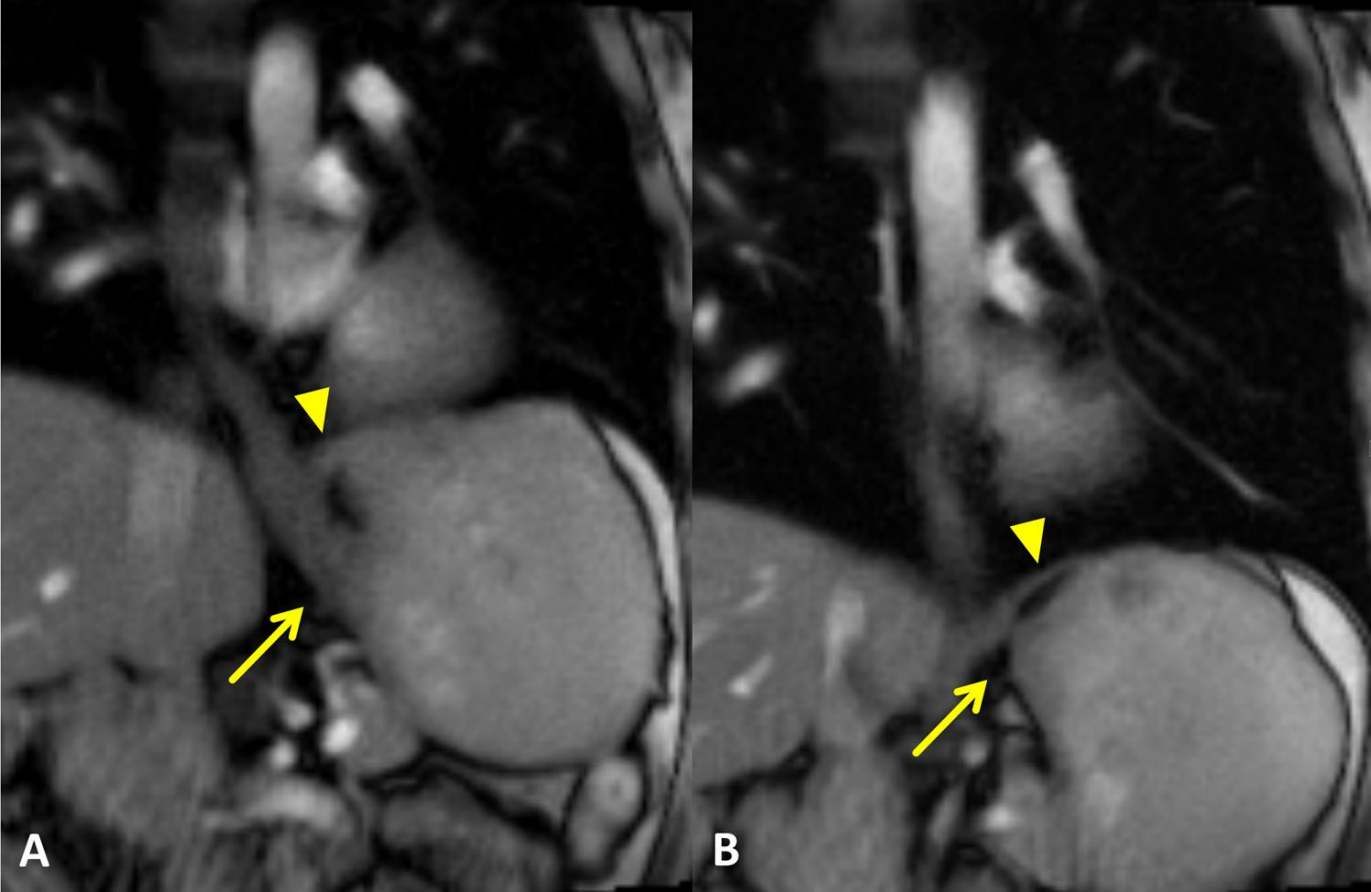
Real-time MRI of the EGJ at rest (**A**) and during Valsalva (**B**) in a patient with EGJ type III on HRM. Real-time MRI films at rest revealed a physiologic positioning of the EGJ (arrow) below the diaphragm (arrowhead) (**A**). Valsalva maneuver resulted in cranialization of the EGJ without herniation through the diaphragm (**B**). See also supplementary material Video 2

There were no differences between different EGJ types regarding the esophageal diameter (*p* = 0.66), sphincter length (*p* = 0.44), and esophagus–fundus angle at rest and during Valsalva (*p* = 0.47 and *p* = 0.41). The mean esophagus–fundus angle measured 85 ± 31° at rest and increased to 101 ± 36° during Valsalva maneuver. The increase of the esophagus–fundus angle was similar for all three groups. Real-time MRI image examples are provided in Fig. 4 (Video 1) and Fig. 5 (Video 2).

## Discussion

In this study, we compared dynamic real-time MRI parameters of the EGJ with EGJ morphology on HRM. On single-contrast barium X-ray study, the delineation of tertiary activity has usually been defined as non-peristaltic contractions that were often multiple and simultaneous, causing a focal but transient narrowing of the esophageal lumen [19]. However, the relationship between manometric and radiographic EGJ morphology has been poorly explored: although Kahrilas and colleagues suggested that hiatal hernia should be radiographically evident in patients with EGJ type II and III, data comparing imaging findings to modern HRM is sparse [11]. In a previous study, real-time MRI not only demonstrated good correlation with endoscopic findings, but also detected endoscopically occult hiatal hernia in a relevant number of patients [17]. In this study, the prevalence of hiatal hernia was significantly higher in patients with EGJ type II and EGJ type III. Still, real-time MRI films of 20% of patients with EGJ type I were graded as hiatal hernia based on morphologic assessment by both readers. Although EGJ type I is defined as an overlap of the LES and the crural diaphragm, HRM studies have shown that EGJ type I can be detected in patients with hiatal hernia [20]. Therefore, hiatal hernia cannot be ruled out by HRM alone.

Further comparison of the sphincter–diaphragm distances at rest and during Valsalva maneuver revealed relevant mobility of the EGJ. Sphincter–diaphragm distances of patients with EGJ type I and II were close to 0 mm at rest and showed a similar cranialization during Valsalva maneuver of − 5.3 ± 14.8 mm in patients with EGJ type I and − 7.0 ± 21.2 mm in patients with EGJ type II. These findings are in line with long-time HRM measurements: the EGJ morphology of patients migrated between EGJ type I and II within the same study [20]. Moreover, a temporary change of the EGJ morphology has been observed after the consumption of liquid meals [21].

The changes of the sphincter–diaphragm distance highlight the mobility of the EGJ as a functional unit under physiologic conditions. In future studies, a cranialization of the LES of up to 10 mm above the diaphragm should be considered as normal and hiatal hernia should therefore only be diagnosed on MRI films if a clear herniation of the fundus is present.

Previous studies using fluoroscopy for EGJ characterization also found mobility of the EGJ in both healthy volunteers and patients with hiatal hernia [22]. However, the low soft tissue delineation limits the precise visualization of the EGJ on fluoroscopic studies. Previous studies, therefore, did not solely rely on barium swallows, but also employed endoscopically placed clips at the squamocolumnar junction of the EGJ for exact tracking of axial EGJ mobility [20, 22]. The requirement for additional endoscopic clip placement renders this method impractical for clinical routine. Moreover, fluoroscopic studies are no longer recommended for primary evaluation of swallowing events by consensus guidelines [23, 24].

So far, the literature comparing manometry and dynamic MRI studies of swallowing events is limited. A feasibility study by Panebianco and colleagues showed promising results for the evaluation of esophageal motility [25]. In support of this notion, dynamic MRI films of the esophagus successfully visualized secondary motility disorders after fundoplication procedure [15, 26]. Curcic and colleagues also investigated an angle between the lower esophagus and the gastric fundus, similar to the esophagus–fundus angle measurements utilized in this study. Curcic reported that the esophagus–fundus angle was larger in GERD patients compared to those without GERD [27]. Similar to our findings, Curcic and colleagues observed a respiration modulation of the esophagogastric insertion angle [27].

Overall, real-time MRI findings matched EGJ morphology on HRM. Furthermore, real-time MRI films can delineate dynamic changes of the EGJ. Despite these findings, this study has several limitations: first, the retrospective nature of this descriptive study and consensus reading imposes bias. Second, optimal comparison of HRM and real-time MRI studies would require a parallel acquisition of MRI films and HRM pressure plots. However, most HRM systems are not built for recording in magnetic fields. Another limitation is the measurability of imaging parameters: the sphincter length and the esophagus–fundus angle at rest and during Valsava maneuver could not be assessed in a relevant number of patients. The limited measurability of these parameters can usually be attributed to an incomplete visualization of the EGJ on MRI films, especially in patients with a distorted EGJ due to hiatal hernia. The application of the sphincter length and esophagus–fundus angle for the characterization of the EGJ is, therefore, limited. Moreover, further studies are required to understand the implications of EGJ mobility for the detection and diagnosis of gastroesophageal reflux. These studies should also further investigate the addition of alternative positioning such as right decubitus position to improve visualization und assessment of the EGJ. While the results of this study suggest that a certain degree of mobility should be considered physiologic, the absence of a healthy control and the number of included patients limit the generalizability of our results.

## Conclusion

The EGJ is a complex anatomic structure that displays high mobility under physiologic conditions. The high temporal resolution of real-time MRI is a non-invasive imaging modality that can visualize dynamic changes of the EGJ during swallowing events.

## Supplementary Information

Below is the link to the electronic supplementary material.

Supplementary Video 1 Real-time MRI of the EGJ at rest (A) and during Valsalva (B and C) in a patient with EGJ type I on HRM. At rest, the EGJ is clearly positioned below the diaphragm, confirming the EGJ morphology on HRM. However, the EGJ moved above the diaphragm during Valsalva maneuver and formed a small hiatal hernia. Repetitive Valsalva maneuver resulted in a relevant size increase of the hiatal hernia (MP4 45343 KB)

Supplementary Video 2 Real-time MRI of the EGJ at rest and during Valsalva in a patient with EGJ type III on HRM. Real-time MRI films at rest revealed a physiologic positioning of the EGJ (arrow) below the diaphragm. Valsalva maneuver resulted in cranialization of the EGJ without herniation through the diaphragm (MP4 18651 KB)

## Abbreviations

EGD: Esophagogastroduodenoscopy
EGJ: Esophagogastric junction
GERD: Gastroesophageal reflux disease
HRM: High-resolution manometry
LES: Lower esophageal sphincter
PPI: Proton pump inhibitor
TLESR: Transient lower esophageal sphincter relaxation
NE: Not evaluable
